# Mathematical Prediction Models for Sentinel Node Status in Early-Stage Breast Cancer: Protocol for a Systematic Review

**DOI:** 10.2196/82523

**Published:** 2026-03-23

**Authors:** Justin James, Kirti Mehta, Mohammadali Ahmadipour, Tegan Ormston, Emily Schembri, Michael Law, Shomik Sengupta, Christobel Saunders

**Affiliations:** 1 Eastern Health Melbourne, Victoria Australia; 2 Eastern Health Clinical School Faculty of medicine, nursing and health sciences Monash University Melbourne, Victoria Australia; 3 Deakin University Melbourne Australia; 4 Universtiy of Melbourne Melbourne Australia

**Keywords:** breast cancer, sentinel node, nomograms, systematic review, artificial intelligence, AI

## Abstract

**Background:**

The status of the axilla remains a significant prognostic factor and influences adjuvant systemic and locoregional treatment choices in early-stage breast cancer (EBC). Sentinel node (SN) biopsy continues to be the preferred technique for establishing axillary nodal status in clinically node-negative EBC. A multivariable prediction model with adequate accuracy and generalizability has been explored as a potential alternative to SN.

**Objective:**

This systematic review aims to evaluate the predictive performance, methodological quality, and risk of bias associated with the available mathematical models (MMs), excluding artificial intelligence (AI)–based models, for predicting SN status in patients with EBC.

**Methods:**

A systematic search will be conducted across PubMed, Cochrane CENTRAL, and Embase to identify studies reporting the development of an SN status prediction model. Only studies that report SN status using mathematical modeling techniques will be included. Two independent reviewers will screen the search results and extract data from the included articles. The primary outcome of this systematic review is to evaluate the methodological adequacy and generalizability of individual MMs and compare the reported predictive performances of methodologically robust MMs. The secondary objective is to identify key predictive factors contributing to SN status prediction in MMs. A narrative synthesis of all the included studies will be undertaken. The details of this protocol are accessible on PROSPERO, where it was registered on January 23, 2025. Ethics approval is not required for this study because only published data will be analyzed.

**Results:**

Funding for this review was obtained in 2022. The literature search was completed on December 15, 2023, and screening began in December 2023. Data extraction and assessment using the Prediction Model Risk of Bias Assessment Tool was completed by December 2025, with synthesis planned for March 2026. Of the 3458 screened records, 122 (3.5%) were selected for data extraction. Results will be prepared for submission for a peer review and publication in mid-2026.

**Conclusions:**

This review will provide a consolidated evaluation of non–machine learning MMs for predicting SN status in EBC. By clarifying the predictive performance and methodological quality of traditional statistical approaches, the findings will serve as a benchmark against which emerging AI-based tools can be compared. This review is also expected to identify predictors that consistently contribute to accurate modeling, informing the development of future statistical and AI-enhanced prediction tools.

**Trial Registration:**

PROSPERO CRD42025637632; https://www.crd.york.ac.uk/PROSPERO/view/CRD42025637632

**International Registered Report Identifier (IRRID):**

DERR1-10.2196/82523

## Introduction

### Background

Sentinel node (SN) biopsy (SNB) is the procedure of choice for assessing the axilla in all patients presenting with clinically node-negative early-stage breast cancer (EBC) [1]. Information on nodal status obtained through SNB contributes to prognostication and can influence the selection of adjuvant systemic and locoregional treatment options [2]. However, as more systemic treatment decisions rely on tumor biology, SN status information is becoming increasingly redundant for many patient subgroups [3]. Importantly, SNB has not demonstrated any proven therapeutic benefit [4]. Moreover, SNB has been shown to adversely affect the quality of life after EBC treatment [5]. Therefore, excluding SNB from the EBC treatment protocol could lead to substantial improvements in workflow efficiency and cost-effectiveness. The accuracy of radiological evaluations of the axilla, combined with the significant impact of tumor biology on systemic and locoregional therapy decisions, has prompted many practitioners to explore alternatives to SNB [4,6-8].

The overall false-negative rate (FNR) of SNB is 8.4% (range 0%-29%), according to the American Society of Clinical Oncology expert panel [9]. It was hypothesized that a reliable prediction model could replace SNB if its accuracy (specifically its FNR and negative predictive value) was comparable to that of SNB [10]. Consequently, many researchers have attempted to predict SN status using preoperative data, and some of these SN status prediction models are well known and have been validated [11,12].

Recent trials and international consensus statements have further accelerated interest in axillary de-escalation for selected patients with clinically node-negative EBC. Randomized studies, such as the Sentinel Node versus Observation After Axillary Ultrasound (SOUND) and Intergroup-Sentinel-Mamma (INSEMA) trials, have demonstrated that omitting SNB in carefully selected low-risk populations is oncologically safe and associated with reduced morbidity [13,14]. Reflecting this evolving evidence base, axillary management recommendations from professional societies, including the American Society of Clinical Oncology, have progressively incorporated principles of de-escalation for selected patients [1,15].

All de-escalation approaches inherently accept low-volume axillary disease that will not be confirmed or excluded in de-escalated patients. Importantly, the St. Gallen International Breast Cancer Consensus has acknowledged that contemporary de-escalation strategies implicitly accept an FNR of approximately 10% to 15% [16].

Historically, nomogram-based mathematical models (MMs) for predicting SN status were not adopted for routine clinical decision-making because their predictive performance was considered insufficient to replace SNB [17]. However, as the paradigm shifts toward accepting low-volume axillary disease, nomograms may assume a new role in aiding patient selection for axillary de-escalation [18,19].

Additionally, more recent studies using advanced modeling approaches—particularly artificial intelligence (AI), radiomics, and genomic data—have reported substantially higher discriminatory performance, with area under the receiver operating characteristic curve values frequently exceeding 0.80 [20-22]. The number of these models has surged recently and has led to optimism toward the development of a noninvasive alternative to SNB [23].

Ideally, a prediction model should adequately report all components of the study so that the results can be interpreted correctly and the model’s applicability in a real-world clinical setting can be inferred. Structured and complete reporting, in accordance with the TRIPOD (Transparent Reporting of a Multivariable Prediction Model for Individual Prognosis or Diagnosis) statement and the PROBAST (Prediction Model Risk of Bias Assessment Tool), is desirable for all prediction model studies [24,25]. Such comprehensive reporting will help replicate and validate the reported models and combine the results in a meta-analysis. Given the significant differences in the reporting parameters, modified standards (TRIPOD+AI and PROBAST+AI) for reporting studies of AI-based models (AIM) have been proposed [26,27].

Therefore, a comprehensive review of currently available models attempting to predict SN status is critical for assessing their quality, transparency, usability, methodological rigor, and risk of bias. This will aid clinicians looking to use a model-based selection strategy for axillary de-escalation. As the parameters used to determine the robustness of prediction models differ between the 2 types—MMs and AIMs—it is challenging to review them together and compare their results within a single systematic review. Although newer AIMs are claimed to be more effective at predicting SN status, MMs remain widely available and could be adopted as decision aids. Moreover, understanding the strengths and limitations of existing MMs will provide a benchmark against which emerging AI-based or hybrid prediction tools can be compared. Identifying critical variables that consistently contribute to SN status prediction in these traditional MMs will also be helpful for future research. These variables may then be selected for the development of more advanced models based on newer modeling techniques.

### Objectives

The primary aim of this study is to systematically review available MMs for predicting SN status in patients with clinically node-negative EBC undergoing primary surgery. We will evaluate the methodological adequacy, suitability, and generalizability of individual MMs and compare the reported predictive performances of methodologically robust MMs. We will conduct a narrative synthesis of robust MMs suitable for clinical use and identify key predictive factors influencing SN status prediction in these MMs.

## Methods

### Overview

This systematic review was designed to meet the reporting standards recommended by TRIPOD Systematic Reviews and Meta-Analyses [28]. The protocol was prepared in accordance with the PRISMA-P (Preferred Reporting Items for Systematic Reviews and Meta-Analyses Protocols) 2015 statement [29] and was registered with PROSPERO (CRD42025637632) on January 23, 2025.

Articles describing primary research aimed at developing multivariable models to predict SN status in patients with EBC undergoing primary surgery were eligible for inclusion. Eligible studies were required to include at least 2 predictors, use any statistical or mathematical modeling methodology, and be published up to December 15, 2023. MMs are defined as statistical, regression-based, or probabilistic modeling techniques that estimate the association between preoperative clinicopathological variables and SN status using prespecified mathematical functions. Included model types are logistic regression models, generalized linear models, generalized additive models, Bayesian statistical models, point-based or rule-based scoring systems derived from statistical modeling, and nomograms derived from these techniques. These models share the following characteristics: (1) prespecified functional form, (2) estimation via statistical inference, and (3) interpretability based on coefficients or explicit equations.

This time frame for including studies was selected to achieve the most comprehensive range of articles that reflect various strategies for predicting SN status. We will include studies of any design and data source, if the predicted outcome is SN status in a population with a clinically node-negative EBC. Further details about the inclusion criteria are provided in Textbox 1.

**Inclusion and exclusion criteria for the study.**

**Inclusion criteria**

- Study design: primary report on development of mathematical models, with or without validation, based on data from experimental (randomized controlled trials) or observational (cohort, case control, and case cohort) studies
- Variables: clinical, radiological, histopathological, or any other measured variables
- Outcome: axillary node status (micrometastasis or macrometastasis) identified using any of the axillary assessment methods (sentinel node biopsy, axillary dissection, axillary sampling, or a combination of these methods) and any of the standard histopathological or immunohistochemical methods
- Population: all or a subgroup of patients with clinically node-negative early-stage breast cancer undergoing surgical primary axillary assessment before any systemic treatment
- Modeling: mathematical modeling techniques including but not restricted to regression-based approaches
- Data source: registry data, hospital and other databases, or clinical trial data

**Exclusion criteria**

- Predictive factor studies: articles describing predictive factors without developing a prediction model
- Validation studies: articles describing the validation of an existing model on new data
- Outcome: models predicting further nodes after a positive sentinel node biopsy and N2 status
- Population: models predicting nodal status after neoadjuvant systemic therapy or in patients with in situ breast cancer
- Modeling: models based on artificial intelligence and machine learning techniques
- Article type and quality: secondary research, conference abstracts, non-English publications, and papers without available full text

Articles will be excluded if they report prediction models developed using machine learning (ML) or AI techniques. These exclusion approaches include but are not limited to (1) decision tree–based methods and ensemble algorithms (eg, classification and regression trees, random forests, Adaptive Boosting, and Extreme Gradient Boosting), (2) support vector machines, (3) neural networks and deep learning models, (4) k-nearest neighbor classifiers, (5) naïve Bayes classifiers, and (6) any nonparametric or high-dimensional modeling approaches in which the functional form is learned algorithmically rather than prespecified. Hybrid mathematical ML approaches will also be excluded unless the ML component is used solely for variable preselection and the final prediction model is purely mathematical. These approaches rely on automated pattern recognition or feature learning and therefore fall within the scope of AI and ML rather than traditional mathematical modeling. We will exclude studies focused on predictive factors, secondary research, conference abstracts, or studies lacking full-text availability in English. Further details on the exclusion criteria are provided in Textbox 1.

A systematic literature search will be conducted in 3 major publicly available electronic medical databases—PubMed, Embase, and Cochrane CENTRAL—to identify all eligible articles published until December 15, 2023. To maximize completeness and minimize the risk of missing eligible studies, we plan to supplement database searches with both backward and forward citation screening. The combination of targeted database searching with citation tracking is expected to retrieve all relevant, high-quality mathematical prediction models; therefore, no additional gray literature sources or trial registries will be searched.

The search strategy was built using keywords related to breast cancer (ie, “breast malignancy” and “breast neoplasm”), SN status (ie, “sentinel node” and “lymph node”), and prediction modeling (ie, “nomograms” and “predictive models”). We selected 10 articles that met our eligibility criteria. A trial run of our search confirmed that it retrieved all 10 chosen studies. The final search strategy is presented in the Multimedia Appendix 1.

Study records, including titles and abstracts, will be imported into Covidence (Veritas Health Innovation) [30], a web-based collaboration platform that streamlines the production of systematic and other literature reviews. It tracks and archives all activities throughout the reviewing process. Once eligible studies are identified, full-text articles will be uploaded into the program for screening and data extraction using a customized template. Two authors (JJ and MA) will independently screen titles and abstracts to identify eligible studies according to the criteria and subsequently review the full texts of potentially relevant studies. If necessary, any disagreements between the reviewers will be resolved by consensus or through consultation with additional investigators (CS and KM). The study flow will be illustrated in a PRISMA (Preferred Reporting Items for Systematic reviews and Meta-Analyses) flowchart.

We will perform double data extraction for all included articles. One of the 2 reviewers (MA and TO) will extract data from each article using a predefined data extraction template, while the lead investigator (JJ) will extract data from all included articles and compare the extracted data. If necessary, a third reviewer (CS) will adjudicate any disagreements in data extraction. The data extraction form will be tested on the first 10 studies and revised as needed. If critical data are required, the authors of the articles will be contacted for additional information and clarification.

The lead investigator (JJ) will maintain all data and records, which will be stored on a shared, secure Covidence platform accessible to all investigators. Data extraction will be guided by TRIPOD adherence criteria, PROBAST, and CHARMS (Checklist for Critical Appraisal and Data Extraction for Systematic Reviews of Prediction Modelling Studies). Table 1 presents the main categories of the extracted data.

**Table 1:** Extracted data categories and their definitions.

Data category	Definition
Study design	Type of study (eg, model development, temporal validation, and geographic validation), methodological design (retrospective, prospective, and registry based), and year of publication
Setting	Geographic location, clinical setting (hospital, multicenter, and registry), and period of data collection
Study population and data source	Eligibility criteria, recruitment method, source of data (hospital records, registry, and cohort study), and population characteristics relevant to model applicability
Patient characteristics	Baseline clinicopathological features (eg, age distribution; tumor size; histology; grade; estrogen receptor, progesterone receptor, and human epidermal growth factor receptor 2 status; and lymphovascular invasion)
Sample size	Number of participants included for model development, (if applicable) internal or external validation, and number of outcome events
Outcome	Definition of sentinel lymph node positivity (eg, macrometastasis >2 mm), method of outcome assessment (SNB^a^ with ALND^b^ and SNB without ALND), and event rate
Predictors	Candidate predictors examined, final predictors retained in the model, definitions of predictors, and handling of missing data
Internal validation	Method and sample used for internal validation (eg, bootstrap, cross validation, and train-test split), and internal performance metrics
External validation	Presence and type of external validation (temporal, geographic, and independent cohort) and characteristics of the validation dataset
Predictive performance measures	Measures of model discrimination (eg, area under the receiver operating characteristic curve), calibration (eg, calibration slope, intercept, and calibration plot), and classification metrics (eg, sensitivity, specificity, positive predictive value, and negative predictive value)

^a^SNB: sentinel node biopsy.

^b^ALND: axillary lymph node dissection.

The form will include instructions for reviewers on assessing the methodological and analytic steps used to develop the model and extracting the relevant statistical parameters presented in the articles. Predictors will be identified from the main article and its associated supplemental files. We will list all the predictors (1) examined as candidates, (2) tested in the model, and (3) included in the final model. The final data extraction form is presented in Multimedia Appendix 2.

The risk of bias in individual studies will be assessed using the PROBAST tool. Before beginning the formal risk-of-bias assessment, all reviewers will complete a calibration exercise using the PROBAST tool on a sample of 3 to 5 included studies. Differences in the interpretation of signaling questions and domain-level judgments will be discussed, and decision rules will be refined to ensure consistency across reviewers. Two reviewers will independently assess the risk of bias for each included study using the PROBAST tool (Multimedia Appendix 3). Disagreements at the signaling question, domain, or overall judgment level will be resolved through discussion. If consensus cannot be reached, a third senior reviewer will adjudicate. Agreement rates will be monitored, and additional calibration will be undertaken if needed. Results from the PROBAST assessment will be fully integrated into the narrative synthesis. Studies judged to be at high overall risk of bias will be highlighted, and their findings will be interpreted with caution. Patterns of bias across domains (participants, predictors, outcomes, and analysis) will be summarized descriptively. Where appropriate, studies at low risk of bias will be prioritized when interpreting the evidence base. No statistical pooling is planned; therefore, PROBAST will primarily guide the qualitative interpretation of study validity and the confidence placed in individual models.

We will conduct a narrative synthesis of the extracted data. Descriptive statistics and visual plots will be used to summarize the data. Categorical data on reporting, methodological practices, and risk of bias will be presented as counts and percentages. The distribution of continuous data, such as sample size and the number of predictors, will be assessed and reported using the mean and SD for normally distributed data and the median with the IQRs for nonnormally distributed data.

The risk-of-bias assessment will be summarized and graphically illustrated for each PROBAST domain, along with an overall risk-of-bias judgment. Results will be stratified by study type (model development only, model development with internal validation, and model development with external validation). Meta-bias will not be investigated, and the strength of the body of evidence will not be assessed in this study.

Protocol amendments will be listed and made available on the PROSPERO registration. Each amendment will include the date, description, and rationale.

### Ethical Considerations

Ethics approval is not required for this study as only deidentified data will be analyzed.

### Dissemination of Findings

The findings of this systematic review will be published in an open-access journal and shared at various scientific conferences.

## Results

**Figure 1:**
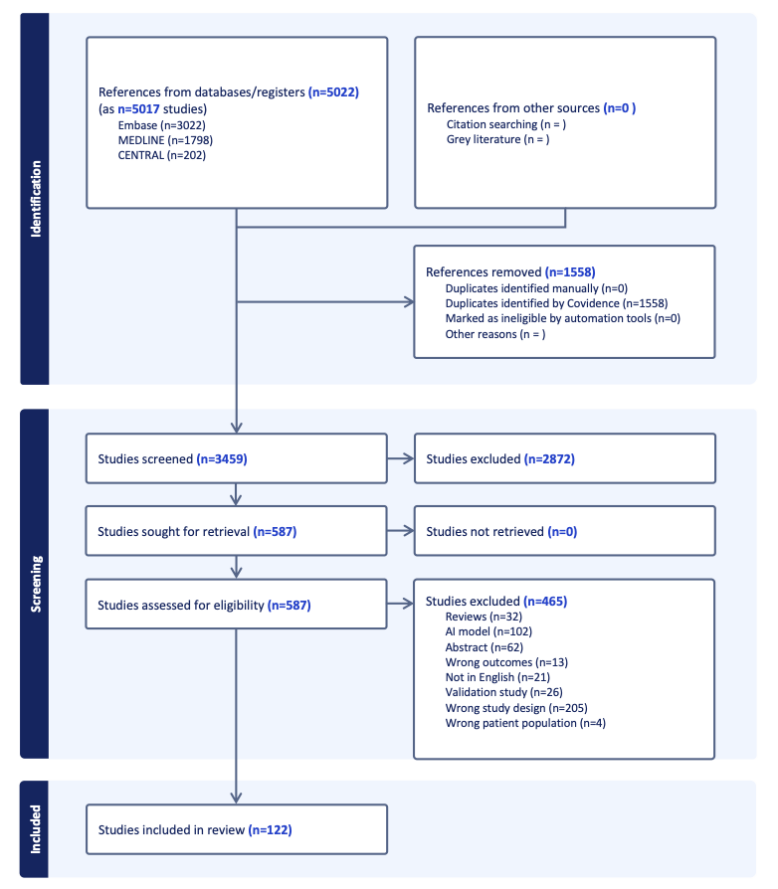
PRISMA (Preferred Reporting Items for Systematic Reviews and Meta-Analyses) flowchart showing the progress of data extraction. AI: artificial intelligence.

Funding for this review was obtained in 2022. The literature search was completed on December 15, 2023, and screening began in December 2023. Data extraction and PROBAST assessment were completed by December 2025, with synthesis planned in March 2026. As of manuscript submission, 3458 records have been screened, and 122 (3.5%) full-text articles were selected for data extraction (Figure 1). Data extraction is ongoing, and the results are expected to be prepared for submission by mid-2026.

## Discussion

### Anticipated Findings

This systematic review is expected to provide the first consolidated synthesis of MMs developed to predict SN status in patients with EBCs. By focusing on MMs, this review will clarify the performance, methodological quality, and potential clinical utility of these traditional statistical models, which rely on routinely collected clinicopathological variables. Therefore, this review fills a gap by examining the evidence base for interpretable, equation-driven prediction tools that promise to aid critical decision-making in the management of EBCs.

A major strength of this protocol is the use of clear operational definitions that distinguish MMs from AIMs, thereby enabling reproducible screening and reducing misclassification. The structured data extraction framework, predefined eligibility criteria and decision rules, and planned use of PROBAST to assess risk of bias across all relevant domains enhance methodological rigor. Supplementing the electronic database searches with forward and backward citation screening increases the likelihood of capturing older or less consistently indexed regression-based models.

The findings of this review will consolidate the strengths and benefits of traditional statistical modeling based on routinely measured clinicopathological variables. By establishing a comprehensive benchmark of model performance and methodological quality, this review will provide a standard against which the performance of emerging AI-based prediction tools can be compared. The synthesis is expected to help determine whether statistical models remain adequate for predicting SN status or whether AI approaches offer meaningful improvements. It will inform clinicians about appropriate models for use with patient groups of interest and in clinical scenarios in which they intend to apply the model. Furthermore, by identifying predictors that consistently contribute to accurate statistical models, this review may guide the selection of key variables for inclusion in future AIMs or hybrid prediction models, supporting the development of efficient, interpretable, and clinically practical tools.

This systematic review is intended to provide a methodological and performance benchmark for MMs of SN status rather than to evaluate their clinical implementation or long-term oncological impact. By focusing exclusively on model development studies, this review will summarize predictive performance, reporting quality, and risk of bias and describe the clinical settings and patient subpopulations (eg, biologically defined subgroups or specific diagnostic pathways) for which individual models were developed. These contextual details will be incorporated into the narrative synthesis to inform the interpretation of model applicability and generalizability.

The findings are expected to support comparison with emerging AIMs or hybrid prediction models, all of which should undergo transparent development, internal validation, rigorous external validation, and prospective testing. As axillary de-escalation strategies ultimately aim to demonstrate oncological safety rather than acceptable FNRs alone, any prediction model intended to guide omission of SNB would require evaluation in randomized controlled trials to establish effects on recurrence and survival. However, such downstream clinical evaluation lies beyond the remit of this review.

By design, this review excludes AIMs that can limit direct comparison across all contemporary modeling techniques. Restricting the search to 3 major databases may omit models published in nontraditional biomedical journals, although citation tracking mitigates this risk. Heterogeneity in study populations, predictor definitions, modeling strategies, and reporting standards may limit comparability between included studies and preclude meta-analysis.

### Conclusions

This systematic review will synthesize and critically appraise MMs predicting SN status in EBCs. By applying rigorous eligibility criteria, structured data extraction methods, and formal risk-of-bias assessment using PROBAST, this review will provide a clear and comprehensive evaluation of the current landscape of statistical prediction tools. The findings will help define the role of mathematical modeling in contemporary axillary decision-making, establish a benchmark for comparison with AI-based approaches, and identify essential predictors that consistently contribute to nodal status prediction, thereby informing future model development and validation.

## Appendix

Multimedia Appendix 1 Search strategy.

Multimedia Appendix 2 Data extraction template.

Multimedia Appendix 3 Quality assessment template.

## Abbreviations

AI: artificial intelligence
AIM: artificial intelligence–based model
CHARMS: Checklist for Critical Appraisal and Data Extraction for Systematic Reviews of Prediction Modelling Studies
EBC: early-stage breast cancer
FNR: false-negative rate
INSEMA: Intergroup-Sentinel-Mamma
ML: machine learning
MM: mathematical model
PRISMA: Preferred Reporting Items for Systematic Reviews and Meta-Analyses
PRISMA-P: Preferred Reporting Items for Systematic Reviews and Meta-Analyses Protocols
PROBAST: Prediction Model Risk of Bias Assessment Tool
SOUND: Sentinel Node versus Observation After Axillary Ultrasound
SN: sentinel node
SNB: sentinel node biopsy
TRIPOD: Transparent Reporting of a Multivariable Prediction Model for Individual Prognosis or Diagnosis

## Data Availability

The extraction framework and the extracted data may be made available on request to support transparency and future methodological research.
