# Supplementary material for: Mathematical Prediction Models for Sentinel Node Status in Early-Stage Breast Cancer: Protocol for a Systematic Review
Source: JMIR Res Protoc. 2026 Mar 23;15:e82523. doi: 10.2196/82523 (PMC13054220; doi:10.2196/82523)
Supplement: Multimedia Appendix 1 [file resprot_v15i1e82523_app1.docx]

## Appendix 1: Search Strategy

### Pubmed Search Strategy

Draft of search strategy for Pubmed database search for “systematic review of mathematical models for predicting sentinel node status in patients with early breast cancer undergoing primary surgery”.

Search date: 15 December 2023

1. Breast Neoplasms/
2. (breast* adj4 (cancer* or neoplasm* or carcinoma* or tumor* or tumour*)).ti,ab,kf.
3. (mammary* adj4 (cancer* or neoplasm* or carcinoma* or tumor* or tumour*)).ti,ab,kf.
4. 1 or 2 or 3
5. nomograms/
6. Models, Statistical/
7. Logistic Models/
8. nomogram*.ti,ab,kf.
9. ((predict* or prognos*) adj4 model*).mp.
10. ((predict* or prognos*) adj4 scor*).mp.
11. (statistic* adj2 model*).mp.
12. (scor* adj2 system*).mp.
13. (Logistic* adj2 Model*).mp.
14. 5 or 6 or 7 or 8 or 9 or 10 or 11 or 12 or 13
15. (axilla* adj2 node*).mp.
16. Lymph Nodes/ or Sentinel Lymph Node/
17. Sentinel Lymph Node Biopsy/
18. lymph node*.ti,ab,kf.
19. sentinel node*.ti,ab,kf.
20. 15 or 16 or 17 or 18 or 19
21. 4 and 14 and 20

### Cochrane Central Search strategy

Draft of search strategy for “Cochrane Central Register of Controlled trials” search for “systematic review of mathematical models for predicting sentinel node status in patients with early breast cancer undergoing primary surgery”.

Search date: 15 December 2023

1. Breast Neoplasms/
2. (breast* adj4 (cancer* or neoplasm* or carcinoma* or tumor* or tumour*)).ti,ab,kf.
3. (mammary* adj4 (cancer* or neoplasm* or carcinoma* or tumor* or tumour*)).ti,ab,kf.
4. 1 or 2 or 3
5. nomograms/
6. Models, Statistical/
7. Logistic Models/
8. nomogram*.ti,ab,kf.
9. ((predict* or prognos*) adj4 model*).mp.
10. ((predict* or prognos*) adj4 scor*).mp.
11. (statistic* adj2 model*).mp.
12. (scor* adj2 system*).mp.
13. (Logistic* adj2 Model*).mp.
14. 5 or 6 or 7 or 8 or 9 or 10 or 11 or 12 or 13
15. (axilla* adj2 node*).mp.
16. Lymph Nodes/ or Sentinel Lymph Node/
17. Sentinel Lymph Node Biopsy/
18. lymph node*.ti,ab,kf.
19. sentinel node*.ti,ab,kf.
20. 15 or 16 or 17 or 18 or 19
21. 4 and 14 and 20

### Embase Search Strategy

Draft of search strategy for “Embase Classic +Embase” search for “systematic review of mathematical models for predicting sentinel node status in patients with early breast cancer undergoing primary surgery”.

Search date: 15 December 2023

1. Breast tumour/
2. (breast* adj4 (cancer* or neoplasm* or carcinoma* or tumor* or tumour*)).ti,ab,kf.
3. (mammary* adj4 (cancer* or neoplasm* or carcinoma* or tumor* or tumour*)).ti,ab,kf.
4. 1 or 2 or 3
5. nomograms/
6. Models, Statistical/
7. Logistic Models/
8. nomogram*.ti,ab,kf.
9. ((predict* or prognos*) adj4 model*).mp.
10. ((predict* or prognos*) adj4 scor*).mp.
11. (statistic* adj2 model*).mp.
12. (scor* adj2 system*).mp.
13. (Logistic* adj2 Model*).mp.
14. 5 or 6 or 7 or 8 or 9 or 10 or 11 or 12 or 13
15. (axilla* adj2 node*).mp.
16. Lymph Nodes/ or Sentinel Lymph Node/
17. Sentinel Lymph Node Biopsy/
18. lymph node*.ti,ab,kf.
19. sentinel node*.ti,ab,kf.
20. 15 or 16 or 17 or 18 or 19
21. 4 and 14 and 20
