# Supplementary material for: Mathematical Prediction Models for Sentinel Node Status in Early-Stage Breast Cancer: Protocol for a Systematic Review
Source: JMIR Res Protoc. 2026 Mar 23;15:e82523. doi: 10.2196/82523 (PMC13054220; doi:10.2196/82523)
Supplement: Multimedia Appendix 2 [file resprot_v15i1e82523_app2.docx]

## Appendix 2: Data extraction template

# General information

**Study ID**

**Title**

Title of paper / abstract / report that data are extracted from

**Lead author contact details**

**Country in which the study conducted**

1. United States
2. UK
3. Canada
4. Australia
5. Unknown
6. Other

**Other country**

**Number of centers**

**Location/ Centre where the participants are recruited from/ study is conducted**

**Setting**

1. Secondary hospital
2. Tertiary hospital/ referral hospital
3. Unknown
4. Others

**Other study setting description**

# Characteristics of included studies

**Aim of study**

**Study Type**

1. Developing, no external validation
2. Developing with external validation
3. Validating only
4. Validating and Updating
5. Predictive factor identification only without model development
6. Other

**Describe other study type**

**Name of Nomogram**

**Study design**

1. Cohort study
2. Other

**Describe "other" study design**

**Source of data**

1. Cohort
2. Others
3. Unknown

**Describe "other" source data**

**Data collection type**

1. Prospective (e.g Follows patients over time)
2. Retrospective (e.g uses existing patient medical records)
3. Combined
4. Others
5. Unknown

**Describe other data collection type**

**Start date**

**End date**

**Study funding sources**

**Possible conflicts of interest for study authors**

# Characteristics of the study population (Participants)

**Population description**

**Study population**

1. All breast cancers
2. All female breast cancers
3. Subgroup of all female breast cancers
4. Others
5. Unknown

**Description of "other" study population**

**Subgroup included**

1. Age group
2. Another patient characteristic
3. Tumour size
4. Tumour histology
5. Others
6. Not applicable
7. Unknown

**Description of the "Other" subgroup included**

**Complete Description of the subgroup**

**Inclusion criteria**

**Exclusion criteria**

1. DCIS
2. Invasive lobular cancer
3. Tumour size not available
4. Sarcoma and other tumours
5. Male breast cancer
6. Multifocal cancers
7. Incomplete data
8. Others
9. Unknown

**Exclusion criteria "others" description**

**Method of recruitment of participants**

1. Consecutive patients
2. Non-consecutive patients
3. Random
4. Unknown
5. Other

**Other method of recruitment of participants**

**Participant description if any**

**Adjuvant treatment received if any at the time.**

1. No treatment
2. Endocrine treatment
3. Chemotherapy
4. Radiotherapy
5. Other
6. Unknown

**Describe other treatment or intervention received by the study cohort**

**Baseline Population Characteristics**

HER2 IHC interpretation

A result of 0 is negative.

A result of 1+ is also negative.

A result of 2+ is considered equivocal (uncertain).

A result of 3+ is positive.

|  | **Value** |
| --- | --- |
| **Mean age or Median age** |  |
| **% of T3 or above Tr size** |  |
| **% IDC, NOS** |  |
| **% of ILC** |  |
| **%of ER positive** |  |
| **% of HER2 positive** |  |
| **% of LVI positive** |  |
| **% of node positivity (Macromet only)** |  |

# Characteristics of the Outcome

**Definition of outcome**

**Definition of outcome**

1. One or more macro metastasis
2. One or micro or macro metastasis
3. One or more ITC, micro or macro metastasis
4. Undefined metastasis
5. Others

**Nodal status assessment method**

1. sentinel node biopsy
2. Axillary dissection
3. Axillary sampling
4. Others
5. Unknown

**Method of histological assessment**

1. Single section H&E (conventional pathology)
2. Serial section H&E
3. IHC
4. Any of the above
5. Unknown
6. Others

**Was the same outcome definition (and method for measurement) used in all patients?**

1. Yes
2. No
3. Unknown
4. Others

**Type of outcome**

1. single
2. combined endpoints
3. Other

**Was the outcome assessed without knowledge of the candidate predictors ?**

1. Yes
2. No
3. Unknown
4. Others

# Candidate predictors

**Type of predictors**

1. Clinical
2. Standard pathological
3. Standard radiological
4. Special radiological (MRI, PET, CT)
5. Special histological including histological sybtyping
6. Radiomics
7. Genomics
8. Others

**Other type of predictor: describe**

**Total number of candidate predictors examined**

Total number of potential predictors (variables)on which the data is collected/ available. Also, please list them in the following field

**Age**

1. Included in the final model (Examined as a candidate predictor,Tried in the model)
2. Examined as a candidate, tried in the model, did not use in the model
3. Examined but not tried in the model
4. Did not examine as a candidate predictor
5. Unknown
6. Other

**Menopausal status**

1. Included in the final model (Examined as a candidate predictor,Tried in the model)
2. Examined as a candidate, tried in the model, did not use in the model
3. Examined but not tried in the model
4. Did not examine as a candidate predictor
5. Unknown
6. Other

**Screen detected or symptomatic**

1. Included in the final model (Examined as a candidate predictor,Tried in the model)
2. Examined as a candidate, tried in the model, did not use in the model
3. Examined but not tried in the model
4. Did not examine as a candidate predictor
5. Unknown
6. Other

**The lesion is palpable or not**

1. Included in the final model (Examined as a candidate predictor,Tried in the model)
2. Examined as a candidate, tried in the model, did not use in the model
3. Examined but not tried in the model
4. Did not examine as a candidate predictor
5. Unknown
6. Other

**Location of the tumour in the breast**

1. Included in the final model (Examined as a candidate predictor,Tried in the model)
2. Examined as a candidate, tried in the model, did not use in the model
3. Examined but not tried in the model
4. Did not examine as a candidate predictor
5. Unknown
6. Other

**Tumour size**

1. Included in the final model (Examined as a candidate predictor,Tried in the model)
2. Examined as a candidate, tried in the model, did not use in the model
3. Examined but not tried in the model
4. Did not examine as a candidate predictor
5. Unknown
6. Other

**Tumour Grade**

1. Included in the final model (Examined as a candidate predictor,Tried in the model)
2. Examined as a candidate, tried in the model, did not use in the model
3. Examined but not tried in the model
4. Did not examine as a candidate predictor
5. Unknown
6. Other

**Histological subtype (IDC, ILC, Special type)**

1. Included in the final model (Examined as a candidate predictor,Tried in the model)
2. Examined as a candidate, tried in the model, did not use in the model
3. Examined but not tried in the model
4. Did not examine as a candidate predictor
5. Unknown
6. Other

**LVI**

1. Included in the final model (Examined as a candidate predictor,Tried in the model)
2. Examined as a candidate, tried in the model, did not use in the model
3. Examined but not tried in the model
4. Did not examine as a candidate predictor
5. Unknown
6. Other

**Peri neural invasion**

1. Included in the final model (Examined as a candidate predictor,Tried in the model)
2. Examined as a candidate, tried in the model, did not use in the model
3. Examined but not tried in the model
4. Did not examine as a candidate predictor
5. Unknown
6. Other

**Multifocality**

1. Included in the final model (Examined as a candidate predictor,Tried in the model)
2. Examined as a candidate, tried in the model, did not use in the model
3. Examined but not tried in the model
4. Did not examine as a candidate predictor
5. Unknown
6. Other

**Oestrogen receptor status**

1. Included in the final model (Examined as a candidate predictor,Tried in the model)
2. Examined as a candidate, tried in the model, did not use in the model
3. Examined but not tried in the model
4. Did not examine as a candidate predictor
5. Unknown
6. Other

**Progesterone receptor status**

1. Included in the final model (Examined as a candidate predictor,Tried in the model)
2. Examined as a candidate, tried in the model, did not use in the model
3. Examined but not tried in the model
4. Did not examine as a candidate predictor
5. Unknown
6. Other

**HER2 status**

1. Included in the final model (Examined as a candidate predictor,Tried in the model)
2. Examined as a candidate, tried in the model, did not use in the model
3. Examined but not tried in the model
4. Did not examine as a candidate predictor
5. Unknown
6. Other

**TILs**

1. Included in the final model (Examined as a candidate predictor,Tried in the model)
2. Examined as a candidate, tried in the model, did not use in the model
3. Examined but not tried in the model
4. Did not examine as a candidate predictor
5. Unknown
6. Other

**Ki 67**

1. Included in the final model (Examined as a candidate predictor,Tried in the model)
2. Examined as a candidate, tried in the model, did not use in the model
3. Examined but not tried in the model
4. Did not examine as a candidate predictor
5. Unknown
6. Other

**Molecular subtype (Luminal, HER2 enriched, TNBC)**

1. Included in the final model (Examined as a candidate predictor,Tried in the model)
2. Examined as a candidate, tried in the model, did not use in the model
3. Examined but not tried in the model
4. Did not examine as a candidate predictor
5. Unknown
6. Other

**Special stains**

1. Included in the final model (Examined as a candidate predictor,Tried in the model)
2. Examined as a candidate, tried in the model, did not use in the model
3. Examined but not tried in the model
4. Did not examine as a candidate predictor
5. Unknown
6. Other

**Tumour to nipple distance**

1. Included in the final model (Examined as a candidate predictor,Tried in the model)
2. Examined as a candidate, tried in the model, did not use in the model
3. Examined but not tried in the model
4. Did not examine as a candidate predictor
5. Unknown
6. Other

**Tumour to skin distance**

1. Included in the final model (Examined as a candidate predictor,Tried in the model)
2. Examined as a candidate, tried in the model, did not use in the model
3. Examined but not tried in the model
4. Did not examine as a candidate predictor
5. Unknown
6. Other

**Radiologically abnormal nodes**

1. Included in the final model (Examined as a candidate predictor,Tried in the model)
2. Examined as a candidate, tried in the model, did not use in the model
3. Examined but not tried in the model
4. Did not examine as a candidate predictor
5. Unknown
6. Other

**USS features of tumour**

1. Included in the final model (Examined as a candidate predictor,Tried in the model)
2. Examined as a candidate, tried in the model, did not use in the model
3. Examined but not tried in the model
4. Did not examine as a candidate predictor
5. Unknown
6. Other

**USS features of axillary node**

1. Included in the final model (Examined as a candidate predictor,Tried in the model)
2. Examined as a candidate, tried in the model, did not use in the model
3. Examined but not tried in the model
4. Did not examine as a candidate predictor
5. Unknown
6. Other

**MRI features of the primary or axilla**

1. Included in the final model (Examined as a candidate predictor,Tried in the model)
2. Examined as a candidate, tried in the model, did not use in the model
3. Examined but not tried in the model
4. Did not examine as a candidate predictor
5. Unknown
6. Other

**PETscan features of the primary or axilla**

1. Included in the final model (Examined as a candidate predictor,Tried in the model)
2. Examined as a candidate, tried in the model, did not use in the model
3. Examined but not tried in the model
4. Did not examine as a candidate predictor
5. Unknown
6. Other

**CT Features of the primary or axilla**

1. Included in the final model (Examined as a candidate predictor,Tried in the model)
2. Examined as a candidate, tried in the model, did not use in the model
3. Examined but not tried in the model
4. Did not examine as a candidate predictor
5. Unknown
6. Other

**Contrast USS features of the primary or axilla**

1. Included in the final model (Examined as a candidate predictor,Tried in the model)
2. Examined as a candidate, tried in the model, did not use in the model
3. Examined but not tried in the model
4. Did not examine as a candidate predictor
5. Unknown
6. Other

**USS Radiomics**

1. Included in the final model (Examined as a candidate predictor,Tried in the model)
2. Examined as a candidate, tried in the model, did not use in the model
3. Examined but not tried in the model
4. Did not examine as a candidate predictor
5. Unknown
6. Other

**MMG Radiomics**

1. Included in the final model (Examined as a candidate predictor,Tried in the model)
2. Examined as a candidate, tried in the model, did not use in the model
3. Examined but not tried in the model
4. Did not examine as a candidate predictor
5. Unknown
6. Other

**Tomosynthesis 3 D mammogram Radiomics**

1. Included in the final model (Examined as a candidate predictor,Tried in the model)
2. Examined as a candidate, tried in the model, did not use in the model
3. Examined but not tried in the model
4. Did not examine as a candidate predictor
5. Unknown
6. Other

**CT Radiomics**

1. Included in the final model (Examined as a candidate predictor,Tried in the model)
2. Examined as a candidate, tried in the model, did not use in the model
3. Examined but not tried in the model
4. Did not examine as a candidate predictor
5. Unknown
6. Other

**MRI Radiomics**

1. Included in the final model (Examined as a candidate predictor,Tried in the model)
2. Examined as a candidate, tried in the model, did not use in the model
3. Examined but not tried in the model
4. Did not examine as a candidate predictor
5. Unknown
6. Other

**PET Radiomics**

1. Included in the final model (Examined as a candidate predictor,Tried in the model)
2. Examined as a candidate, tried in the model, did not use in the model
3. Examined but not tried in the model
4. Did not examine as a candidate predictor
5. Unknown
6. Other

**Genomic predictors**

1. Included in the final model (Examined as a candidate predictor,Tried in the model)
2. Examined as a candidate, tried in the model, did not use in the model
3. Examined but not tried in the model
4. Did not examine as a candidate predictor
5. Unknown
6. Other

**Machine learning of histology slides**

1. Included in the final model (Examined as a candidate predictor,Tried in the model)
2. Examined as a candidate, tried in the model, did not use in the model
3. Examined but not tried in the model
4. Did not examine as a candidate predictor
5. Unknown
6. Other

**"Other" candidate predictors not listed above**

Please list all other discrete candidate predictors examined irrespective of the final state.

**Timing of predictor measurement**

1. Before surgery
2. After surgery
3. Both
4. Variable
5. Others
6. Unknown

**Were predictors assessed blinded for outcome, and for each other**

1. yes
2. no
3. Unknown
4. Others

# Predictors in the Model

**Method for selection of predictors for inclusion/exclusion in multivariable modelling**

1. All candidate predictors
2. Predictors found significant on univariate analysis (unadjusted association with the outcome)
3. Missing data
4. Predetemined variables
5. Other
6. Unknown

**Other method for selection of variables for inclusion in the model**

**Total number of predictors tried in the model**

The model may not try all examined variables. Usually, only those variables found to be significant on univariate analysis are tried in the model.

**Total number of predictors in the final model**

**List of predictors in the final model that are not selected appropriately in the previous list of candidate predictors**

You would have already selected many of the predictors in the previous list of candidate predictors appropriately as "included in the final model." If any other candidate predictors are included in the model, please list them here. The total number of predictors already selected and listed here should match the total number entered in the previous field.

# Sample size

**Sample size table Regression models**

|  | **Candidate** | **Participant in the study** | **Participant in the final model** | **Internal validation** | **External validation** |
| --- | --- | --- | --- | --- | --- |
| **Total number** |  |  |  |  |  |
| **Outcome number** |  |  |  |  |  |
| **Events per predictor** |  |  |  |  |  |

**Sample size table for AI models**

|  | **Candidate** | **Participant in the study** | **Participant in the final model** | **Taining set** | **Validation set** | **Test set** | **Ext validation** |
| --- | --- | --- | --- | --- | --- | --- | --- |
| **Total number** |  |  |  |  |  |  |  |
| **Outcome number** |  |  |  |  |  |  |  |
| **Events per predictor** |  |  |  |  |  |  |  |

# Missing data

**Number of participants with any missing value (include predictors and outcomes)**

**Number of participants with missing data for each predictor**

**Handling of missing data**

1. Excluded from the model
2. Multiple imputation
3. Others
4. Unknown

**Other method of handling of missing data**

# MODEL DEVELOPMENT

**Type of model.**

1. Linear regression
2. Logistic regression
3. Neural net work
4. Decision tree
5. AI
6. Others
7. Combined
8. Unknown

**Type of model "other" description**

**Methods used to transform predictors prior to inclusion in the multivariable analysis**

1. Continuous
2. Linear
3. Non-linear transformations
4. Categorized
5. Splines
6. Other
7. Unknown

**Other Methods used to transform predictors prior to inclusion in the multivariable analysis**

**Method for selection of predictors during multivariable modelling**

In statistical modelling, a full model refers to a model that includes all potential predictors or independent variables. Backward selection is where you start with the full model and sequentially remove the least significant variables until the best subset of variables is identified. Forward selection is the opposite approach; you start with no variables and add them one by one, testing at each step if the new variable significantly improves the model.

1. Enter method/ full model approach
2. Stepwise (unspecified)
3. Stepwise backward elimiation
4. Stepwise forward selection
5. Both direction stepwise selection
6. Others
7. Unknown

**Other method for selection of predictors during multivariable modelling**

**Criteria used for elimination of predictor**

1. Akaike or Bayesian Information Criterion
2. Nominal p-value
3. Change in the model’s c-index
4. Others
5. Unknown

**Other criteria used for elimination of predictor**

**Shrinkage of predictor weights or regression coefficients**

1. no shrinkage
2. uniform shrinkage
3. penalized estimation
4. Others
5. Unknown

**Other shrinkage method**

**Model equation**

# MODEL PERFORMANCE

**Calibration measures**

Assessing the calibration of a logistic regression model is crucial to ensure that the predicted probabilities align well with the actual outcomes. Here are some common calibration measures and methods:

1. Calibration Plot (Reliability Diagram): This is a graphical method where predicted probabilities are plotted against observed outcomes. Ideally, the points should lie on the 45-degree line, indicating perfect calibration1.

2. Hosmer-Lemeshow Test: This statistical test divides the data into deciles based on predicted probabilities and compares the observed and expected frequencies in each decile. A significant test result indicates poor calibration2.

3. Brier Score: This is a measure of the mean squared difference between predicted probabilities and the actual outcomes. Lower Brier scores indicate better calibration3.

4. Logarithmic Loss (Log Loss): This metric measures the performance of a classification model where the prediction input is a probability value between 0 and 1. Lower log loss values indicate better calibration3.

5. Calibration Slope and Intercept: The calibration slope is obtained by regressing the observed outcomes on the predicted probabilities. A slope of 1 indicates perfect calibration. The intercept should ideally be 03.

6. Expected Calibration Error (ECE): This metric quantifies the difference between predicted probabilities and actual outcomes, averaged over all predictions. Lower ECE values indicate better calibration3.

7. Integrated Calibration Index (ICI): This is a summary measure that integrates the absolute differences between predicted probabilities and observed outcomes over the entire range of predicted probabilities3.

These measures help in understanding how well the logistic regression model’s predicted probabilities match the actual outcomes, ensuring the model’s reliability and accuracy.

1. Calibration plot
2. Calibration slope
3. Hosmer-Lemeshow test
4. Other
5. Unknown

**Other calibration measure**

**Calibration Value**

**confidence interval**

**Discrimination measures**

Assessing the discrimination of a logistic regression model is essential to determine how well the model distinguishes between different outcome classes. Here are some common discrimination measures:

1. Area Under the Receiver Operating Characteristic Curve (AUC-ROC): This is one of the most widely used measures. The ROC curve plots the true positive rate (sensitivity) against the false positive rate (1-specificity) at various threshold settings. The AUC represents the probability that a randomly chosen positive instance is ranked higher than a randomly chosen negative instance1.

2. Gini Coefficient: This is derived from the AUC-ROC and is calculated as (2 \times \text{AUC} - 1). It ranges from 0 (no discrimination) to 1 (perfect discrimination)1.

3. Sensitivity and Specificity: Sensitivity (or recall) measures the proportion of actual positives correctly identified by the model, while specificity measures the proportion of actual negatives correctly identified2.

4. Precision-Recall Curve and Area Under the Precision-Recall Curve (AUC-PR): This curve plots precision (positive predictive value) against recall (sensitivity). The AUC-PR is particularly useful for imbalanced datasets where the number of positive instances is much smaller than the number of negative instances2.

5. Discrimination Slope: This is the difference between the mean predicted probabilities for the positive and negative classes. A higher discrimination slope indicates better discrimination2.

6. Net Reclassification Index (NRI): This measure evaluates the improvement in risk prediction by comparing the predicted probabilities of two models. It assesses how well the new model reclassifies subjects into higher or lower risk categories compared to the old model2.

7. Integrated Discrimination Improvement (IDI): Similar to NRI, IDI measures the improvement in model discrimination by comparing the difference in predicted probabilities between models for events and non-events2.

These measures help in evaluating how effectively a logistic regression model can distinguish between different outcome classes, ensuring its practical utility in predictive tasks.

1. C-Statistic/AUC
2. D-Statistic
3. log-rank
4. Other
5. Unknown

**Other discrimination measure**

**Discrimination Value:**

**confidence interval**

**Type of Validation methods**

Internal validation in logistic regression models is essential to ensure that your model performs well and generalizes within the dataset it was trained on. Here are some common techniques:

1. Cross-Validation

. K-Fold Cross-Validation: The dataset is divided into ( k ) subsets (folds). The model is trained on ( k-1 ) folds and tested on the remaining fold. This process is repeated ( k ) times, with each fold used exactly once as the test set. The results are averaged to provide a performance estimate1.

• Leave-One-Out Cross-Validation (LOOCV): A special case of k-fold cross-validation where ( k ) equals the number of observations. Each observation is used once as a test set while the rest serve as the training set1.

2. Bootstrap Validation

• Bootstrapping: This involves repeatedly sampling with replacement from the dataset to create multiple bootstrap samples. The model is trained on each sample and tested on the out-of-sample data (data not included in the bootstrap sample). This helps estimate the model’s performance and its variability12.

3. Split-Sample Validation

• Training and Testing Split: The dataset is randomly split into two parts: a training set and a testing set. The model is trained on the training set and evaluated on the testing set. This method is straightforward but can be less reliable if the dataset is small3.

Practical Example

For a logistic regression model predicting disease presence based on patient data:

1. K-Fold Cross-Validation: Split the data into 10 folds. Train the model on 9 folds and test on the 10th. Repeat this 10 times and average the results.

2. Bootstrap Validation: Create 1000 bootstrap samples. Train the model on each sample and test on the data not included in the sample. Calculate the average performance metrics.

These methods help ensure that your logistic regression model is robust and not overfitting to the training data.

1. Bootstrapping
2. Cross validation
3. Unknown
4. Other

**Other type of validation method**

**classification measures**

Assessing the classification performance of a logistic regression model involves several key metrics. Here are the most common ones:

1. Accuracy: This is the ratio of correctly predicted instances to the total instances. It gives a general idea of how well the model is performing but can be misleading for imbalanced datasets1.

2. Precision: Also known as Positive Predictive Value, precision is the ratio of true positive predictions to the total predicted positives. It indicates how many of the predicted positive cases are actually positive1.

3. Recall (Sensitivity or True Positive Rate): This is the ratio of true positive predictions to the total actual positives. It measures the model’s ability to identify positive instances1.

4. F1 Score: The F1 score is the harmonic mean of precision and recall. It provides a single metric that balances both precision and recall, especially useful for imbalanced datasets1.

5. Specificity (True Negative Rate): This is the ratio of true negative predictions to the total actual negatives. It measures the model’s ability to identify negative instances1.

6. Receiver Operating Characteristic (ROC) Curve and Area Under the ROC Curve (AUC-ROC): The ROC curve plots the true positive rate against the false positive rate at various threshold settings. The AUC-ROC represents the probability that a randomly chosen positive instance is ranked higher than a randomly chosen negative instance1.

7. Confusion Matrix: This is a table that summarizes the performance of a classification model by showing the true positives, true negatives, false positives, and false negatives. It provides a comprehensive view of the model’s performance1.

8. Logarithmic Loss (Log Loss): This metric measures the performance of a classification model where the prediction input is a probability value between 0 and 1. Lower log loss values indicate better performance1.

These measures help in evaluating different aspects of the model’s performance, ensuring a comprehensive assessment of its classification capabilities.

1. sensitivity
2. specificity
3. predictive values
4. Overall accuracy
5. net reclassification improvement
6. Net Benefit analysis
7. Other
8. Unknown

**Other classification measure**

**Classification Values:**

**whether a-priori cut points were used**

**Model statistics**

|  | **Value Test** | **Confidence interval Test** | **Value Int validation** | **Confidence interval int validation** | **Value Ext validation** | **Confidence interval Ext validation** |
| --- | --- | --- | --- | --- | --- | --- |
| **Calibration plot** |  |  |  |  |  |  |
| **Calibration slope** |  |  |  |  |  |  |
| **Hosmer-Lemeshow test** |  |  |  |  |  |  |
| **Other Calibration measures** |  |  |  |  |  |  |
| **C-Statistic/AUC** |  |  |  |  |  |  |
| **D-Statistic** |  |  |  |  |  |  |
| **log-rank** |  |  |  |  |  |  |
| **sensitivity** |  |  |  |  |  |  |
| **specificity** |  |  |  |  |  |  |
| **positive predictive values** |  |  |  |  |  |  |
| **negative predictive values** |  |  |  |  |  |  |
| **net reclassification improvement** |  |  |  |  |  |  |
| **net benefit anlysis** |  |  |  |  |  |  |
| **Overall accuracy** |  |  |  |  |  |  |
| **Other statistic 1** |  |  |  |  |  |  |
| **Other statistic 2** |  |  |  |  |  |  |
| **Other statistic 3** |  |  |  |  |  |  |
