# Supplementary material for: Mathematical Prediction Models for Sentinel Node Status in Early-Stage Breast Cancer: Protocol for a Systematic Review
Source: JMIR Res Protoc. 2026 Mar 23;15:e82523. doi: 10.2196/82523 (PMC13054220; doi:10.2196/82523)
Supplement: Multimedia Appendix 3 [file resprot_v15i1e82523_app3.docx]

## Appendix 3: Quality Assessment Template

**D1. Participant: SQ1. Data source appropriateness**

Describe the source of data in sufficient detail to allow an assessment of whether it should produce an appropriate and representative population as claimed.

1. Yes/ Probably yes
2. No/ Probably not
3. Unsure

*Extractors will also be able to add supporting text to justify their judgements*

**D1. Participant: SQ2. Inclusion and exclusion criteria appropriateness**

Were all inclusions and exclusions of participants appropriate?

1. Yes/ Probably yes
2. No/ Probably not
3. Unsure

*Extractors will also be able to add supporting text to justify their judgements*

**D1. Risk of bias introduced by participant**

Low risk of bias: If the answer to all signalling questions is "Yes" or "Probably yes," then the risk of bias can be considered low. If ≥1 of the answers is "No" or "Probably no," the judgment could still be "Low risk of bias", but specific reasons should be provided as to why the risk of bias can be considered low.

High risk of bias: If the answer to any signalling questions is "No" or "Probably no," there is a potential for bias, except if defined at low risk of bias above.

Unclear risk of bias: If relevant information is missing for some of the signalling questions and none of the signalling questions is judged to put this domain at high risk of bias.

1. High
2. Low
3. Unsure

*Extractors will also be able to add supporting text to justify their judgements*

**D2. Predictors: SQ1. Consistency of assessment**

Were predictors defined and assessed in a similar way for all participants?

1. Yes/ Probably yes
2. No/ Probably not
3. Unsure

*Extractors will also be able to add supporting text to justify their judgements*

**D2. Predictors: SQ2. Adequacy of blinding**

Were predictor assessments made without knowledge of outcome data?

1. Yes/ Probably yes
2. No/ Probably not
3. Unsure

*Extractors will also be able to add supporting text to justify their judgements*

**D2. Predictors: SQ3. Completeness**

Are all predictors available when the model is intended to be used?

1. Yes/ Probably yes
2. No/ Probably not
3. Unsure

*Extractors will also be able to add supporting text to justify their judgements*

**D2. Risk of bias introduced by predictors**

Low risk of bias: If the answer to all signalling questions is "Yes" or "Probably yes," then the risk of bias can be considered low. If ≥1 of the answers is "No" or "Probably no," the judgment could still be "Low risk of bias", but specific reasons should be provided as to why the risk of bias can be considered low.

High risk of bias: If the answer to any signalling questions is "No" or "Probably no," there is a potential for bias, except if defined at low risk of bias above.

Unclear risk of bias: If relevant information is missing for some of the signalling questions and none of the signalling questions is judged to put this domain at high risk of bias.

1. High
2. Low
3. Unsure

*Extractors will also be able to add supporting text to justify their judgements*

**D3. Outcome SQ1 Determination of the outcome (Node positivity)**

Was the outcome (Node positivity) determined appropriately? (SNB or ALND is considered adequate, while other methods are to be evaluated for their merit and described)

1. Yes/ Probably yes
2. No/ Probably not
3. Unsure

*Extractors will also be able to add supporting text to justify their judgements*

**D3. Outcome SQ2 Appropriateness of the "Histological confirmation technique" of the outcome (Node positivity)**

Was the histological confirmation of the outcome (Node positivity) determined appropriately? (Serial section H&E is considered adequate, while other methods are to be evaluated for their merit and described)

1. Yes/ Probably yes
2. No/ Probably not
3. Unsure

*Extractors will also be able to add supporting text to justify their judgements*

**D3. Outcome SQ3, Use of a standard definition of the outcome**

Was a pre-specified or standard outcome definition used? (Macro metastasis is considered the standard outcome, while Micro metastasis and ITC are not)

1. Yes/ Probably yes
2. No/ Probably not
3. Unsure

*Extractors will also be able to add supporting text to justify their judgements*

**D3. Outcome SQ4, Consistent Use of a standard definition of the outcome**

Was the outcome defined and determined in a similar way for all participants?

1. Yes/ Probably yes
2. No/ Probably not
3. Unsure

*Extractors will also be able to add supporting text to justify their judgements*

**D3. Outcome SQ5, Blinding of predictors from outcome measurement**

Was the outcome determined without knowledge of predictor information?

1. Yes/ Probably yes
2. No/ Probably not
3. Unsure

*Extractors will also be able to add supporting text to justify their judgements*

**D3. Risk of bias introduced by outcome measurement**

Low risk of bias: If the answer to all signalling questions is "Yes" or "Probably yes," then the risk of bias can be considered low. If ≥1 of the answers is "No" or "Probably no," the judgment could still be "Low risk of bias", but specific reasons should be provided as to why the risk of bias can be considered low.

High risk of bias: If the answer to any signalling questions is "No" or "Probably no," there is a potential for bias, except if defined at low risk of bias above.

Unclear risk of bias: If relevant information is missing for some of the signalling questions and none of the signalling questions is judged to put this domain at high risk of bias.

1. High
2. Low
3. Unsure

*Extractors will also be able to add supporting text to justify their judgements*

**D4. Analysis. SQ1, Adequate Participant with outcome**

Were there a reasonable number of participants with the outcome?

Yes: If the number of participants with the outcome relative to the number of candidate predictor parameters is ≥20 (EPV ≥20).

No/probably no: if the number of participants with the outcome relative to the number of candidate predictor parameters is < 10 (EPV < 10).

No information: no information on the number of candidate predictor parameters or number of participants with the outcome, such that the EPV cannot be calculated.

1. Yes/ Probably yes
2. No/ Probably not
3. Unsure

*Extractors will also be able to add supporting text to justify their judgements*

**D4 Analysis. SQ2, Handling of continuous and categorical variables**

Were continuous and categorical handled appropriately?

Yes, probably yes: If continuous predictors are not converted into two or more categories when included in the model (i.e., dichotomized or categorized), or if continuous predictors are examined for nonlinearity using, for example, fractional polynomials or restricted cubic splines,

No or probably no: If categorical predictor group definitions do not follow a prespecified method, or if continuous predictors are grouped into two or more categories when included in the model.

No information: There is no information on whether continuous predictors are examined for nonlinearity, and there is also no information on how categorical predictor groups are defined.

1. Yes/ Probably yes
2. No/ Probably not
3. Unsure

*Extractors will also be able to add supporting text to justify their judgements*

**D4 Analysis. SQ3, Were enrolled participants included in the analysis?**

1. Yes/ Probably yes
2. No/ Probably not
3. Unsure

*Extractors will also be able to add supporting text to justify their judgements*

**D4 Analysis. SQ4,Were participants with missing data handled appropriately?**

1. Yes/ Probably yes
2. No/ Probably not
3. Unsure

*Extractors will also be able to add supporting text to justify their judgements*

**D4 Analysis. SQ5, Was selection of predictors based on univariable analysis avoided?**

1. Yes/ Probably yes
2. No/ Probably not
3. Unsure

*Extractors will also be able to add supporting text to justify their judgements*

**D4 Analysis. SQ6, Were relevant model performance measures evaluated appropriately?**

Yes/probably yes: If both calibration and discrimination are evaluated appropriately

No/probably no: If neither calibration nor discrimination is assessed, or if only goodness-of-fit tests, such as the Hosmer-Lemeshow test used for evaluating calibration, are presented, or if classification measures (like sensitivity, specificity, or predictive values) are shown using predicted probability thresholds derived from the available data set.

No information: Neither calibration nor discrimination has been reported, or there are no details on thresholds for estimating classification measures.

1. Yes/ Probably yes
2. No/ Probably not
3. Unsure

*Extractors will also be able to add supporting text to justify their judgements*

**D4 Analysis. SQ7, Were model overfitting, under-fitting, and optimism in model performance accounted for?**

1. Yes/ Probably yes
2. No/ Probably not
3. Unsure

*Extractors will also be able to add supporting text to justify their judgements*

**D4 Analysis. SQ8, Do predictors and their assigned weights in the final model correspond to the results from the reported multivariable analysis**

1. Yes/ Probably yes
2. No/ Probably not
3. Unsure

*Extractors will also be able to add supporting text to justify their judgements*

**D4 Risk of bias introduced by analysis**

Low risk of bias: If the answer to all signalling questions is "Yes" or "Probably yes," then the risk of bias can be considered low. If ≥1 of the answers is "No" or "Probably no," the judgment could still be "Low risk of bias", but specific

reasons should be provided as to why the risk of bias can be considered low.

High risk of bias: If the answer to any signalling questions is "No" or "Probably no," there is a potential for bias, except if defined at low risk of bias above.

Unclear risk of bias: If relevant information is missing for some of the signalling questions and

none of the signalling questions is judged to put this domain at high risk of bias.

1. High
2. Low
3. Unsure

*Extractors will also be able to add supporting text to justify their judgements*

**Overall risk of bias**

Low ROB: If all domains are rated as low risk of bias. (A prediction model developed without any external validation and rated as low risk of bias for all domains should be considered for downgrading to high risk of bias. Such a model can only be deemed low risk of bias if the development is based on a very large dataset and includes some form of internal validation.)

High ROB: If ≥1 domain is judged at high risk of bias.

Unclear ROB: If an unclear risk of bias was noted in ≥1 domain and it was low risk for all other domains.

1. High
2. Low
3. Unsure

*Extractors will also be able to add supporting text to justify their judgements*

Click or tap here to enter text.
